# Composite transcriptome assembly of RNA-seq data in a sheep model for delayed bone healing

**DOI:** 10.1186/1471-2164-12-158

**Published:** 2011-03-24

**Authors:** Marten Jäger, Claus-Eric Ott, Johannes Grünhagen, Jochen Hecht, Hanna Schell, Stefan Mundlos, Georg N Duda, Peter N Robinson, Jasmin Lienau

**Affiliations:** 1 Institute for Medical Genetics, Charité-Universitätsmedizin Berlin, Augustenburgerplatz 1, 13353 Berlin, Germany; 2 Berlin-Brandenburg Center for Regenerative Therapies (BCRT), Charité-Universitätsmedizin Berlin, Augustenburgerplatz 1, 13353 Berlin, Germany; 3 Max Planck Institute for Molecular Genetics, Ihnestrasse 73, 14195 Berlin, Germany; 4 Julius Wolff Institute and Center for Musculoskeletal Surgery, Charité-Universitätsmedizin Berlin, Augustenburgerplatz 1, 13353 Berlin, Germany

## Abstract

**Background:**

The sheep is an important model organism for many types of medically relevant research, but molecular genetic experiments in the sheep have been limited by the lack of knowledge about ovine gene sequences.

**Results:**

Prior to our study, mRNA sequences for only 1,556 partial or complete ovine genes were publicly available. Therefore, we developed a composite *de novo *transcriptome assembly method for next-generation sequence data to combine known ovine mRNA and EST sequences, mRNA sequences from mouse and cow, and sequences assembled *de novo *from short read RNA-Seq data into a composite reference transcriptome, and identified transcripts from over 12 thousand previously undescribed ovine genes. Gene expression analysis based on these data revealed substantially different expression profiles in standard versus delayed bone healing in an ovine tibial osteotomy model. Hundreds of transcripts were differentially expressed between standard and delayed healing and between the time points of the standard and delayed healing groups. We used the sheep sequences to design quantitative RT-PCR assays with which we validated the differential expression of 26 genes that had been identified by RNA-seq analysis. A number of clusters of characteristic expression profiles could be identified, some of which showed striking differences between the standard and delayed healing groups. Gene Ontology (GO) analysis showed that the differentially expressed genes were enriched in terms including *extracellular matrix*, *cartilage development*, *contractile fiber*, and *chemokine activity*.

**Conclusions:**

Our results provide a first atlas of gene expression profiles and differentially expressed genes in standard and delayed bone healing in a large-animal model and provide a number of clues as to the shifts in gene expression that underlie delayed bone healing. In the course of our study, we identified transcripts of 13,987 ovine genes, including 12,431 genes for which no sequence information was previously available. This information will provide a basis for future molecular research involving the sheep as a model organism.

## Background

The sheep is an established model organism for medically relevant research in cardiology [1,2], reproductive medicine [3], respiratory medicine [4,5] and many other fields. The sheep is particularly important in the field of orthopedics, because the dimensions of ovine long bones allow the use of implants designed for application in humans, and the bone mineral composition as well as the metabolic and remodeling rates are similar to those in humans [6-10].

Although bone usually heals spontaneously, failures in bone healing remain an important medical and research challenge. Bone healing is a highly complex regenerative process that is directed by a series of cytokines and growth factors and leads to restoration of skeletal integrity. Despite advances in the field of orthopedic research, our understanding of the molecular mechanisms involved in standard and impaired healing is still limited, and delayed unions and non-unions are still major clinical problems.

A tibial osteotomy healing model in sheep is well established in our laboratory, and its biological and mechanical characteristics have been previously published [11-17]. The model has been used to simulate standard healing by means of the application of a rigid external fixator following osteotomy as well as delayed healing by application of a rotationally unstable external fixator.

To date, molecular research in the sheep has been hampered by the paucity of information about the sheep genome and gene sequences. Therefore, a systematic comparison of the expression characteristics of the transcriptome between a standard and impaired healing osteotomy has not yet been performed. Massively parallel DNA sequencing platforms, widely referred to as "next-generation sequencing" (NGS), are changing the playing field in biomedical research by enabling the comprehensive and relatively inexpensive analysis of genomes and transcriptomes [18-22]. NGS technologies have opened the door to genome scale experiments in organisms that lack comprehensive genome or transcriptome information, making it possible to assemble novel transcripts and identify differential regulation in a single experiment [23,24].

We have previously used EST sequencing to investigate genes differentially expressed in the course of standard bone healing in sheep [25]. In this study, we compare the gene expression profiles of standard and delayed bone healing in the sheep by means of next-generation sequencing and computational analysis of the sheep transcriptome to identify transcripts of 13,987 ovine genes, for 12,431 of which no mRNA sequence was previously available. We identified characteristic clusters of gene expression including several with striking differences between standard and delayed healing, some of which have known roles in the extracellular matrix and skeletal development.

## Results

### Sheep osteotomy model

**Figure 1:**
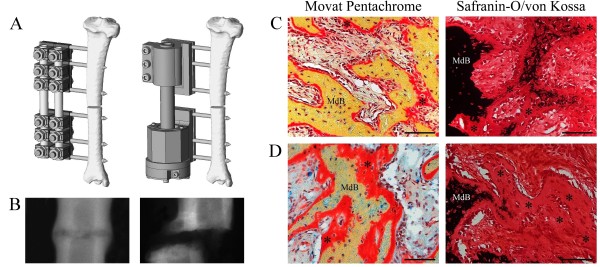
**Sheep Bone Healing Model**. **A **Standard bone healing was investigated in a 3 mm tibial osteotomy model stabilized with a medially mounted rigid external fixator (left). Delayed healing was investigated in a 3 mm tibial osteotomy model stabilized with a medially mounted rotationally unstable (right) external fixator (caudo-lateral view). **B **Cranio-caudal radiographs of the rigid (left) and unstable (right) fixator group 63 days post-operation. Images modified from Schell et al. 2008 [17]. **C **Photomicrographs of representative histological sections of the periosteal bony callus at 14 days from standard healing. Newly formed bone in the periosteal callus covered by a layer of osteoid seam (asterisks, left image) and region of intramembranous bone formation (right image) with mineralization of deposited osteoid (asterisks). **D **Photomicrographs of representative histological sections of the periosteal bony callus at 14 days from delayed healing. Increased amount of osteoid (asterisks) covering the newly formed bone (left image) and less mineralization of deposited osteoid (right image). (C, D) Movat Pentachrome staining (left column), Safranin-Orange/von Kossa staining (right column). MdB: mineralized bone. The scale bars in lower right hand corners are equal to 100 *μ*m.

A total of 63 female Merino mix sheep (2.5 years old) with a mean weight of 72 kg (± 10 kg) received a standardized mid-shaft tibial osteotomy (3 mm gap) stabilized with a monolateral external fixator. Group I (*n *= 31) received a rigid external fixator, which has been previously shown to support standard healing [15,16]. Group II (*n *= 32) was treated with a mechanically critical external fixator, which allowed free rotation through a highly stiff stainless-steel tube set in two tapered roller bearings, thereby producing a distinct delayed healing [17] (Figure 1A). The standard healing group stabilized with the rigid external fixator showed uneventful healing with complete bony bridging by day 42 or 63. In contrast, the delayed healing group stabilized with the rotationally unstable external fixator did not reach union by day 63 (Figure 1B). Calcified histology at day 14 and 21 revealed newly formed bone in the periosteal callus that was covered by a layer of osteoid seam in the control group. Furthermore, in regions of intramembranous bone formation periosteally, mineralization of deposited osteoid was visible (Figure 1C). In contrast, in the delayed healing group, an increased amount of osteoid covering the newly formed bone and less mineralization of deposited osteoid was found at day 14 (Figure 1D).

### Massively parallel sequencing

To date, the use of the sheep to investigate the genetic correlates of bone healing has been limited owing to the lack of ovine genome and transcriptome sequences. The purpose of the current project was therefore to generate an initial sheep transcriptome using next-generation technologies and to perform an exploratory analysis of differential gene expression between standard and delayed healing in the sheep.

**Figure 2:**
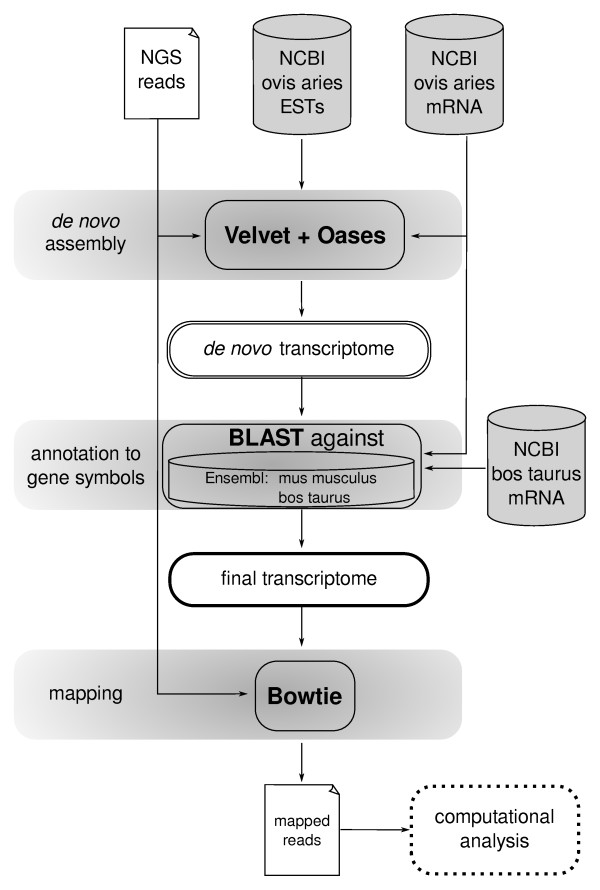
**Bioinformatics workflow**. The figure summarizes computational procedures for assembly, annotation, and mapping of the NGS reads.

We developed a bioinformatics workflow (Figure 2) that would take advantage of ovine sequences where possible and otherwise use homologous sequences from mouse and cow for mapping short reads. We analyzed one flowcell with eight lanes of samples at different time points of standard and delayed healing. Pooled samples were obtained from 5-6 animals each at four different time points (7, 11, 14, 21 days). Each sample was run in a single lane of the flowcell, resulting in 18-27 million 76 bp reads per lane corresponding to 9-14 million unique reads per lane with a total of 177 million reads including 69 million unique reads (Table 1).

**Table 1:** Read counts

lane	reads	unique reads
1	22,145,090	11,682,018
2	23,356,043	12,420,275
3	27,617,415	14,663,522
4	20,234,612	10,168,941
5	24,793,840	12,944,693
6	18,894,344	9,796,924
7	18,788,777	10,383,698
8	21,722,529	12,302,346
all	177,552,650	69,019,744

Lanes 1, 3, 5, and 7 correspond to standard bone healing on days 7, 11, 14, and 21, and lanes 2, 4, 6, and 8 to delayed healing on days 7, 11, 14, and 21.

### De novo transcriptome assembly

Prior to *de novo *assembly the reads from all lanes of the flowcell were pooled and duplicate reads were removed to generate a list with each different sequence represented only once. The best mean read score for a single unique read was preserved. The list of unique reads was then trimmed by removing base calls from the 3' end of the read with Phred (quality) score [26] of 2 or less. A total of 4,599 ovine mRNA genbank entries (corresponding to 1,556 genes) and 325,596 ovine ESTs were downloaded from the NCBI database using the E-Utils [27]. NCBI reference sequences containing one or more ambiguous bases ('N') were removed from further analysis, such that 4,363 mRNA and 294,896 EST sequence files were available for the assembly. *De novo *assembly using the mRNAs, the ESTs, and the uniquified and trimmed reads was then performed using Velvet [28]. Velvet output a total of 830,469 contigs with an average length of 134 bp. Oases is a *de novo *transcriptome assembler designed to produce extended contigs from short read sequencing technologies in the absence of any genomic assembly. It clusters the contigs from a preliminary assembly by Velvet into small groups called loci and uses a de Bruijn graph-based algorithm to construct transcript isoforms [29]. The contigs produced by Velvet were postprocessed using Oases yielding 85,555 loci (gene predictions) comprising a total of 117,594 extended contigs with an average length of 1,374 bp. 56,298 loci exceeded the minimum length threshold of 150 bp and were included in further analysis (Table 2). For each of the 56,298 loci, the contig with the highest Oases confidence score was chosen for further analysis. The average length of these contigs was 956 bp.

**Table 2:** Results of the de novo assembly using Velvet and Oases

Source	Description	number
Velvet	Contigs	830,469
	Average contig length (bp)	134
		
Oases	Extended contigs	117,594
	loci	85,555
	loci > 150 bp	56,298
	loci (quality filtered + annotated)	22,117
	Number of unique mappable sheep genes	13,546
	Average annotated contig length (bp)	1,662
		
NCBI	ovine genbank mRNA entries	4,599
	ovine genes with known mRNA sequence	1,556
	ovine EST entries	325,596
	bovine genbank mRNA entries	43,102
	bovine genes with known mRNA sequence	16,052
		
Assembly	Total sheep genes with known mRNA sequence	13,987

Following all quality control and filtering steps, there were a total of 24,325 mappable genes. For 21,865 of these genes, positive counts were detected in all 8 lanes, and these genes were used for the further analysis of differential expression. A *gene with known mRNA sequence *refers to a gene with a gene symbol for which at least one mRNA sequence was found. Note that mRNA sequence entries assigned to hypothetical genes were not included, and that multiple sequence entries were found for some genes. A *unique mappable sheep gene *refers to a set of one or more Oases loci that could be mapped to a unique gene symbol via BLASTing to sheep, cow, or mouse sequences. The *total number of sheep genes with known RNA sequence *is derived from the union of *de novo *assembled genes and previously sequenced genes, 1,115 of which overlapped.

BLAST was used to identify the gene models by comparing the contig sequences to available sheep, cow, and mouse mRNA sequences. The resulting hits (limited to only the one best matching sequence per query sequence) were filtered for matches with significant *E*-value according to the species being compared and percentage of identical matches meeting the requirements shown in Table 3. In comparison to *Ovis aries*, more reference sequences are available for *Bos taurus *including various splice variants. Therefore, a higher identity cut-off was used to identify cow ortholog transcript models. 22,117 contigs were annotated to a gene symbol using this pipeline. The average length of the annotated contigs was 1,662 bp, and the longest single annotated contig was 21,746 bp long and annotated to dystonin (*DST*). There were 34,181 contigs which could not be assigned to any mouse, cow or sheep transcript, and these were excluded from further analysis.

**Table 3:** Threshold settings used for homolog mapping using Blastx/n

	identical matches	expect value
Blastx (mouse)	≥ 80%	≤ 1^-20^
Blastn (sheep)	≥ 90%	≤ 1^-50^
Blastn (cow)	≥ 97%	≤ 1^-50^

Only Blast matches fulfilling both conditions were considered as true orthologs.

### Read mapping

**Figure 3:**
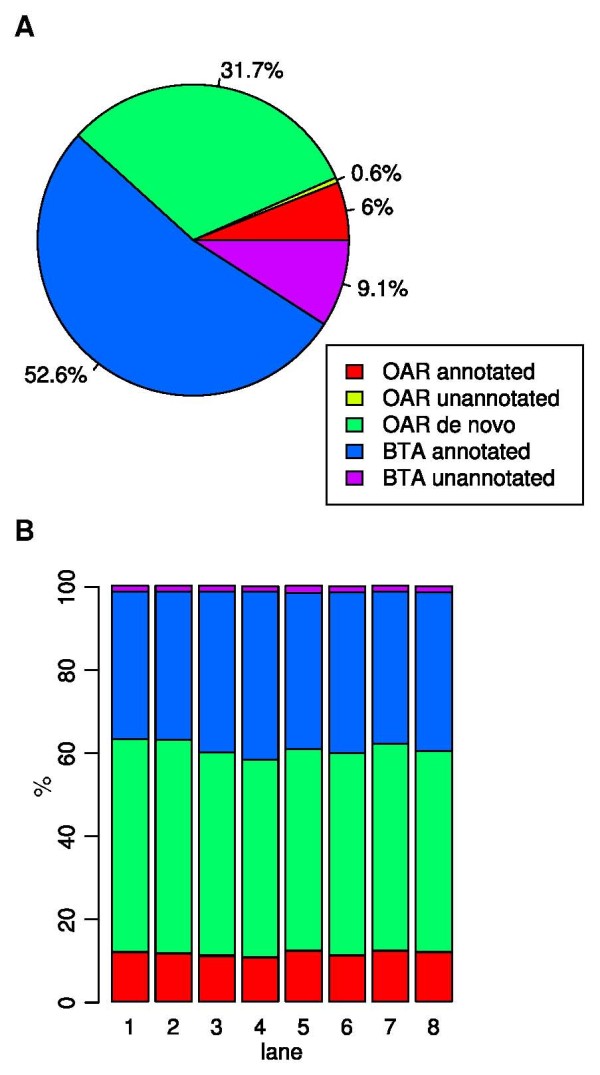
**Composite reference transcriptome assembly and assignment of short read sequences**. **A ***De novo *transcriptome assembly was performed with Velvet and Oases on the basis of mRNA sequence entries from GenBank for sheep and cow as well as the *de novo *assembled contigs from this study. All sequences were used as targets to map the short reads. The pie chart shows the relative proportions of the sequence entries from each of the sources used for the mapping (OAR = *Ovis aries*, BTA = *Bos taurus*). **B **The distribution of the targets that were matched by bowtie for short read mapping are shown. Most reads mapped to the *de novo *transcriptome assembly, but it was possible to map a substantial number of additional reads by use of the *Ovis aries *and *Bos taurus *mRNA sequences. Table 4 displays the exact counts for each lane.

The *de novo *transcriptome was then combined with all available 4,599 ovine mRNA sequence files as well as all 43,102 bovine mRNA sequence files from NCBI for mapping of the short reads with Bowtie [30]. The Bowtie mapping algorithm was used to map short reads with a seed length of 18 and a maximum of three allowed mismatches in the seed. In each lane each read was mapped to a single gene. On average, about 75% of the short reads could be mapped (Table 4). The use of *Bos taurus *transcripts for the mapping substantially increased the number of mappable reads (Figure 3).

**Table 4:** Read counts for the 8 lanes as mapped with Bowtie

				Sheep (%)			Cow (%)	
lane	reads	mapped reads	%	annot	unannot	*de novo*	annot	unannot
1	22,145,090	17,127,532	77.3	11.8	0.1	51.2	35.4	1.5
2	23,356,043	17,964,339	76.9	11.5	0.1	51.4	35.5	1.5
3	27,617,415	17,964,339	76.3	10.8	0.4	48.7	38.6	1.5
4	20,234,612	14,980,730	74.0	10.5	0.1	47.6	40.4	1.3
5	24,793,840	18,367,757	74.1	12.1	0.2	48.4	37.6	1.7
6	18,894,344	13,810,552	73.1	11.0	0.1	48.6	38.7	1.5
7	18,788,777	13,599,338	73.1	12.1	0.2	49.6	36.7	1.4
8	21,722,529	14,276,174	65.7	11.8	0.2	48.2	38.2	1.5

The sections entitled Sheep (*Ovis aries*) and Cow (*Bos taurus*) indicate the percentage of the corresponding target sequences for the mapped reads for each of the eight lanes. See also Figure 3.

### Evaluation of differential gene expression

Each of the lanes corresponded to a pooled sample of 5-6 animals at one time point. As one lane per condition was available, differential expression between conditions was evaluated using the Audic-Claverie method [31,32] in order to enable exploratory analysis. The raw counts of the reads mapped as described above were used for the Audic-Claverie analysis. In addition, RPKM analysis was used to estimate the fold change. Gene expression was compared between the time point 7 days and those at 11, 14, and 21 days for both standard and delayed healing. In addition, each of the individual time points was compared between the standard and the delayed healing groups. A gene was considered to be differentially expressed if the Audic-Claverie *p*-value was < 10 ^-15 ^and the fold change of the normalized (RPKM) expression values was at least 2 in either direction (see also Additional file 1: Supplemental Figure S2). There were 5 genes differentially expressed between standard and delayed healing at day 7, 173 at day 11, 59 at day 14, and 109 at day 21. Within the time course of standard healing, there were 177 genes differentially expressed between day 11 and 7, 265 between day 14 and 7, and 318 between day 21 and 7. Within the time course of delayed healing, there were 136 genes differentially expressed between day 11 and 7, 139 between day 14 and 7, and 259 between day 21 and 7. This corresponded to a total of 884 distinct genes showing differential expression in at least one comparison. Q-PCR analysis of the pooled samples for 26 selected genes confirmed the analysis of the RPKM values (Additional file 1: Supplemental Tables S1-S4 and Figure S3).

### Clustering and GO analysis

**Figure 4:**
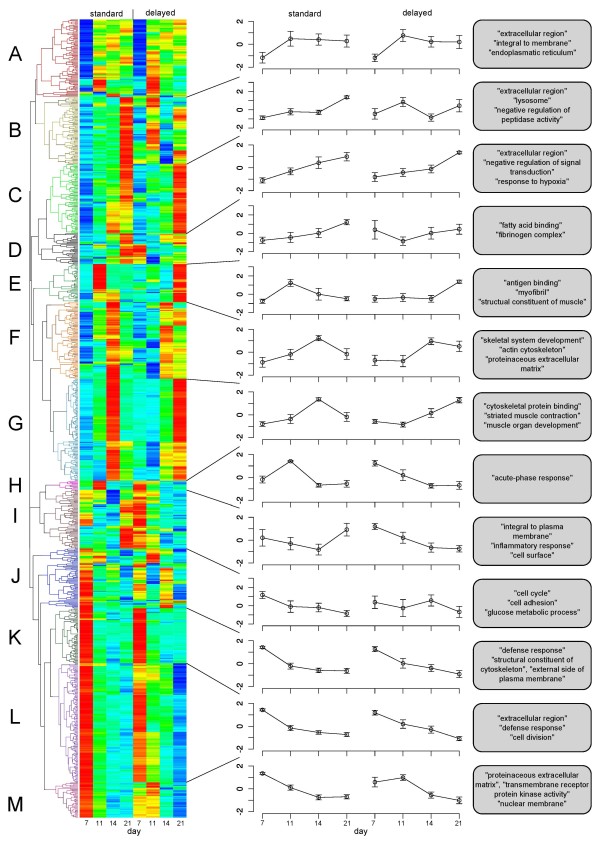
**Gene Clustering**. Cluster of the 884 genes significant in at least one experimental condition between time points using Audic-Claverie testing (p-value < 10 ^-15^) and a fold change of at least 2. The normalized (mean 0, standard deviation 1) RPKM values were calculated separately for standard and delayed healing. We could identify 13 clusters by visual inspection of the heatmap, where blue indicates low and red high expression. For each cluster the normalized relative expression with mean and standard deviation per day and condition were plotted (center) and a maximum of three most significant Gene Ontology terms (right) are shown. The Gene Ontology analysis was performed using the Ontologizer with MGSA (see methods).

Hierarchical clustering was performed to identify groups of genes with similar expression profiles. A total of 13 clusters were identified by visual inspection. Figure 4 shows the expression profile for standard and delayed healing groups. The heat plot on the left displays the expression patterns for all genes. Separate plots are shown for each cluster with the average and standard deviations of the RPKM expression values. There were clusters with relatively minor differences between the standard and delayed healing groups, and several others with marked differences between the groups. Model-based gene set (MGSA) Gene Ontology analysis [33] was performed for each of the clusters, and up to three GO terms with a marginal probability of at least 50% and the highest number of annotated genes are shown (Figure 4). Additionally, MGSA was performed for the entire set of 884 differentially expressed genes (Table 5).

**Table 5:** Model-based gene set analysis

ID	Name	Marginal	Count
GO:0031012	extracellular matrix	0.984	81/299
GO:0006941	striated muscle contraction	0.913	24/54
GO:0043292	contractile fiber	0.847	45/113
GO:0006096	glycolysis	0.680	13/48
GO:0051384	response to glucocorticoid stimulus	0.635	20/79
GO:0008009	chemokine activity	0.583	9/33
GO:0006956	complement activation	0.502	14/34
GO:0042246	tissue regeneration	0.481	9/28

The 884 differentially expressed genes were analyzed as the study set in comparison to a total of 15,343 mapped ovine genes for which a human gene symbol was identified. The column 'marginal' indicates the marginal probability of a term being in the 'active' state, and the column 'count' shows the counts of genes in the study (x) and population (y) sets as x/y.

**Figure 5:**
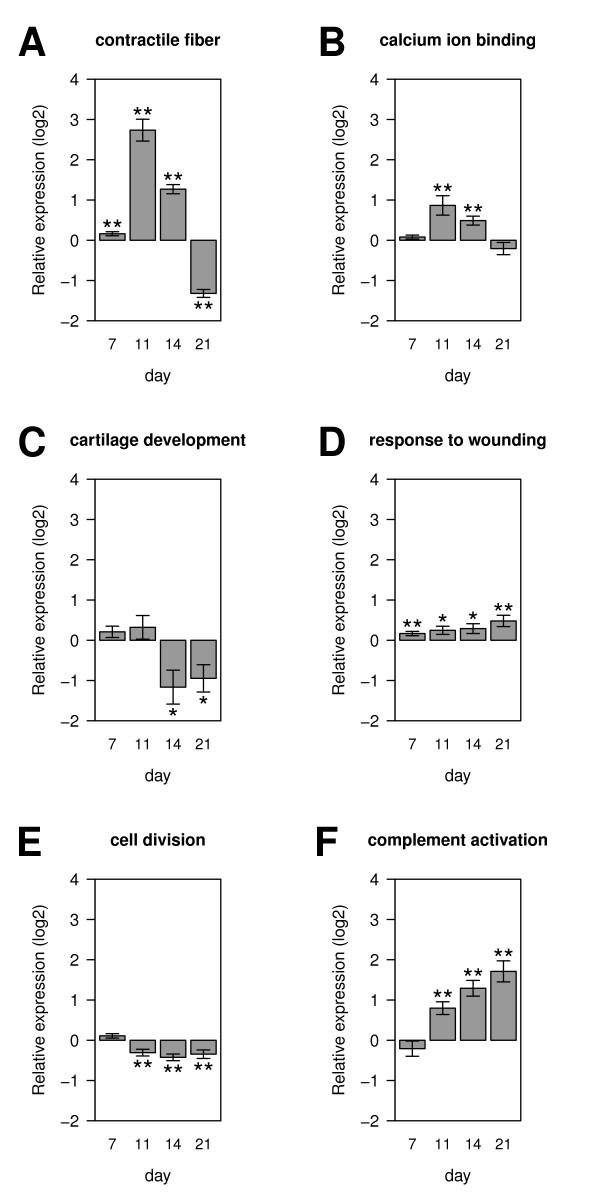
**Differentially expression of genes annotated to selected GO-terms**. **A ***contractile fiber *(GO:0043292), **B ***calcium ion binding *(GO:0005509), **C ***cartilage development *(GO:0051216), **D ***response to wounding *(GO:0009611), **E ***cell division *(GO:0051301), and **F ***complement activation *(GO:0006956). The normalized RPKM values of all significantly differentially expressed genes annotated to the indicated GO terms from standard healing were divided by the corresponding RPKM values from delayed healing. Positive values indicate higher expression levels in standard healing, negative values higher expression levels in delayed healing. A *t*-test against the null-hypothesis that there is no difference between the healing groups was performed (* *p *< 0.05, ** *p *< 0.01).

The GO terms include terms such as *extracellular matrix *and *chemokine activity *with well known roles in skeletal biology and bone healing. 24 of the differentially expressed genes were annotated to *striated muscle contraction*, and 45 to *contractile fiber*. The role of *α*-smooth muscle actin fibroblasts in the contraction of skin wounds is well known; smooth-muscle actin expressing connective tissue cells have also been shown to take part in fracture healing [34], and our results could be a reflection of this phenomenon. Therefore, we investigated all differentially expressed genes annotated to *contractile fiber *(*n *= 45). Most of these genes are localized in clusters E and G (Figure 4) and were more highly expressed in standard healing at day 11 and 14, and more highly expressed in the delayed healing group at day 21, consistent with a delay in the regulation of these genes (Figure 5A). Several of the genes annotated to *striated muscle contraction *and *contractile fiber *overlap with genes annotated to *calcium ion binding *(*n *= 59). These genes were also significantly more highly expressed in standard healing at day 11 and day 14 (Figure 5B) where clear signs of mineralization were shown by histology (Figure 1C). In contrast, differentially expressed genes that were annotated to *cartilage development *(*n *= 10) showed a characteristic upregulation in the latter two time points of delayed healing (Figure 5C). These genes are a subgroup of genes that are annotated to the GO term *skeletal system development*, which was significant in the MGSA analysis of cluster F (Figure 4).

Especially during the early phase of bone healing, fundamental processes such as inflammation are strongly influenced by the mechanical conditions under which bone healing takes place [35,36]. In our experiment, genes annotated to *response to wounding *(*n *= 84) were consistently more highly expressed in the standard healing group (Figure 5D). This term is a parent term of *inflammatory response *(cluster I) and related to *defense response *(cluster K and L). In agreement with the biological observation that the inflammatory phase of bone healing takes place in the first few days [37] most genes annotated to these terms showed the highest expression at day 7 both in standard and in delayed healing. In a similar fashion genes annotated to *cell division *(*n *= 29) showed the highest expression levels at day 7 (cluster L). These genes were consistently more highly expressed in the delayed healing group at the later time points (Figure 5E). Genes involved in *complement activation *were significantly overrepresented in all 884 differentially expressed genes (Table 5) and were from day 11 on significantly more highly expressed in the standard healing group (Figure 5F).

## Discussion

Most bony injuries heal without problems, but there are several conditions under which enhancement of the repair process would be of great benefit to ensure the rapid restoration of skeletal function. Animal models are essential for investigating the different molecular processes underlying bone healing. Several studies have identified molecular differences between standard healing fractures and experimentally induced delayed healing or non-unions [38-40], but these studies have been performed in small-animal models. Currently, investigative tools are still limited for analyses in large-animal models such as sheep and relatively little research has been performed into sheep genetics. However, the sheep model is critical for medical applications because the size of the bone, the loading, and the time to healing are comparable to human fracture healing [6].

A virtual sheep genome has been constructed by mapping ovine contigs obtained by 454 sequencing onto bovine sequences that had been rearranged in sheep order [41-44]. At present, however, only low coverage genomic data is available. The sequences presented in this work will represent a valuable and complementary resource to current efforts to sequence the sheep genome.

In previous studies, we have focused on investigating the expression of specific genes during mechanically induced delayed healing in the sheep osteotomy model compared to standard healing [12,13]. The present study is the first conducting a systematic comparison of the expression characteristics of the transcriptome between a standard and impaired healing osteotomy in a large-animal model. A limitation of our study is the fact that only a single pooled sample could be investigated for each condition. The Audic-Claverie test allows an estimation of the statistical significance of observed differences in the counts of genes that are interpreted as differential expression, but may tend to overestimate significance. For this reason, we applied a stringent *P*-value cutoff (a gene was considered differentially expressed with a Benjamini-Hochberg corrected *P*-value of *p *< 10 ^-15 ^and a fold-change of at least 2-fold). With this proviso, our study has identified a large number of differentially expressed genes corresponding to biological categories that are thought to be most relevant for bone healing. For instance, transcriptome-wide analyses revealed that about 9% (81/884) of the genes found to be differentially expressed during bone healing are annotated to *extracellular matrix*. Some of these ECM genes are typically found in cartilage. Semi-rigid fixation associated with delayed healing results in a larger cartilage component of the callus, which persisted longer [11]. In agreement with this observation, our study showed higher expression of genes related to cartilage formation after mechanically critical fixation with higher instability of the bone fragments. Cytokines play important roles during bone healing and were shown to be significantly overrepresented in the MGSA analysis. We additionally identified a large set of genes annotated to *striated muscle contraction and contractile fiber *that displayed a characteristic shift in delayed bone healing. Genes from these categories have not previously been known to be differentially expressed in bone healing. More research will be required to identify the cell types within the fracture callus that express these genes and to elucidate their functional role. The fact that many genes whose proteins are involved in binding calcium ions are differentially expressed provides leads as to the molecular correlates of the differential mineralization observed in delayed bone healing.

## Conclusions

RNA-Seq is an approach to expression profiling based on next-generation sequencing technologies, whereby a sample of RNA is converted to a library of cDNA fragments attached to adaptors. Individual molecules, with or without amplification, are then sequenced in a high throughput fashion [45]. For model organisms such as the sheep for which relatively few gene sequences have been previously published, RNA-seq allows combined identification of previously unsequenced transcripts together with transcriptome analysis. Prior to this study, partial or complete mRNA sequences corresponding to 1,556 ovine genes were publicly available. In the course of this project, partial or complete transcript sequences were generated for 13,987 ovine genes, corresponding to a nearly nine-fold increase in the number of sheep genes with publicly available sequence information. A FASTA file with sequences of the longest available transcript for each of the 13,987 ovine genes is available as Additional File 2. The short reads have been deposited in NCBI's short read archive.

## Methods

### Surgical procedure

All animal experiments were conducted following national regulations for the care and use of laboratory animals and approved by the local legal representative (Landesamt für Gesundheit und Soziales Berlin: G0127/07, G0172/04). Surgery was performed as described previously [12,17]. The newly generated tissues were harvested at days 7, 11, 14 and 21 after surgery. For all time points the sample size was *n *= 6 for both groups, except for day 21 (group I, *n *= 5; group II, *n *= 6). In the 7 day groups, the tissue formed in the gap was harvested under general anesthesia and the animals were sacrificed 14 days after osteotomy, for comparative analysis of healing after hematoma harvesting which will be reported in another study. In the other groups, tissue harvesting occurred after euthanasia and involved removal of newly formed tissue at the osteotomy site (within the gap and along the periosteal/endosteal surfaces of the bone fragments). Tissue was placed in an RNA stabilization reagent (RNAlater; Qiagen, Hilden, Germany) for storage at -80°C.

### RNA extraction

Total RNA was isolated from the tissues using the RNeasy Maxi Kit (Qiagen) according to the manufacturer's instructions. The concentration of each RNA sample was determined spectrophotometrically and the integrity of all RNA samples was monitored on agarose gels.

### Histology

For histological examination, the callus regions of the explanted tibiae from additional animals of the standard and delayed healing groups euthanized at the day 14 (*n *= 4 each) were sectioned into 3 mm slices in the frontal plane. For calcified histology, histological slices were dehydrated with alcohol and xylol, embedded in methylmetacrylate (Technovit 9100 NEU, Heraeus Kulzer, Germany), cut into 6 *μ*m-thick sections and stained with Movat Pentachrome and Safranin Orange/von Kossa.

### Library preparation and Massively parallel sequencing

In each experimental group total RNA of all samples per time point was pooled prior to library preparation. All libraries were prepared using the mRNA-Seq sample prep Kit (Illumina, San Diego, CA USA) according to the manufacturer's instruction. Clusters were generated with Illumina's v4 Single Read Cluster Generation Kit. Each library was loaded onto one lane of the flow cell at 7 pM concentration. The flow cell was then sequenced on a Genome Analyzer IIx (Illumina) for 76 cycles with v4 sequencing kits following the standard protocol and using SCS v2.6 software.

### Expression profiling and Analysis of Differential Expression

The mapped read counts for each gene were normalized for RNA length and for the total read number in the lane according to reads per kilobase of exon model per million mapped reads (RPKM), which facilitates comparison of transcript levels between samples [45]. The Audic-Claverie method was used to estimate differential expression between standard and delayed bone healing at the same time point as well as for the comparison of different time points within each group. The Audic-Claverie method, which was originally developed for SAGE data, is based on the assumption that the counts of each gene in each of two libraries under comparison follow the same unknown Poisson distribution, and thus allows an estimation of differential expression based on single measurements for two conditions [31]. Fold changes were calculated after quantile normalization of the RPKM values (Additional file 1: Supplemental Figure S1).

### Gene Ontology Analysis

Gene Ontology [46] annotations for the 21,865 sheep gene models were obtained by mapping the gene symbols for the sheep gene models to human gene symbols (*n *= 15,343) using bioMart [47]. GO annotations were available for 13,785 of these genes. Model-based gene set analysis (MGSA) was used to perform Gene Ontology analysis. MGSA analyzes all GO terms at once by embedding them in a Bayesian network, in which gene response is modeled as a function of the activation of the GO terms and probabilistic inference is used to identify the active categories [33]. Analysis was performed using the Ontologizer [48].

### Clustering

For each gene determined to be differentially expressed, normalized RPKM values were transformed to a mean of zero and a standard deviation of one separately for the standard and delayed healing groups (four time points each). The normalized values were then combined into vectors of 8 values per gene. Hierarchical clustering was performed to group genes according to similarity in pattern of gene expression [49].

### Short read sequences

The data from the experiments described in this work are available from the NCBI Sequence Read Archive at http://www.ncbi.nlm.nih.gov/sra under the accession number SRA020182.

### Quantitative PCR (Q-PCR)

After transcription of 1 *μ*g RNA into cDNA (RevertAid H minus cDNA Synthesis Kit, Fermentas) quantitative RT-PCR was performed in MicroAmp optical 384-well plates on ABI Prism 7900 Sequence Detection System in a total volume of 12 *μ*l in each well containing 6 *μ*l of Power SYBR Green PCR Master Mix (Applied Biosystems), 5 *μ*l cDNA (in a 1:50 dilution) and 1 *μ*l primers (0.2 *μ*mol each). For some RNA samples with lower concentrations, higher volumes were reverse transcribed, and the cDNA dilutions were adapted accordingly prior to Q-PCR analysis. For validation of the target genes obtained from RNA-seq equal amounts of cDNA were pooled for each group and time point. Primer pairs were designed to span exon-exon junctions. Primer sequences can be obtained upon request. All samples were run in triplicates in separate tubes to permit the quantification of the target genes' mRNA expression relative to the mean expression of *GTPB1*, *HDAC6 *and *SNRPN*, i.e. three stably and highly expressed genes obtained from the RNA-seq data. Q-PCR result data was exported from the SDS 2.3 software (Applied Biosystems) and further analyzed as described previously [50].

## Authors' contributions

JL, CEO, JH, PNR, GND and SM conceived the study. HS and JL performed the animal experiments. JH designed and performed the RNA-seq experiments. MJ and PNR designed and performed the bioinformatic analysis, and together with CEO analyzed the data. CEO and JG designed, and performed the quantitative RT-PCR experiments. CEO, JL, MJ, and PNR wrote the manuscript. All of the authors contributed to the research design, discussed the results, and commented on the manuscript.

## Supplementary Material

Additional file 1 **Supplementary Information**. A PDF file with Supplementary Figures S1 - S3 and Supplementary Table S1 - S4. Click here for file

Additional file 2 **Ovis aries composite transcriptome**. A FASTA file containing the sequence of the longest transcript of each of the 13,987 ovine gene models. Click here for file
