# Supplementary material for: Composite transcriptome assembly of RNA-seq data in a sheep model for delayed bone healing
Source: BMC Genomics. 2011 Mar 24;12:158. doi: 10.1186/1471-2164-12-158 (PMC3074554; doi:10.1186/1471-2164-12-158)
Supplement: Additional file 1 — Supplementary Information. A PDF file with Supplementary Figures S1 - S3 and Supplementary Table S1 - S4. [file 1471-2164-12-158-S1.PDF]

Supplementary Information

Composite Transcriptome Assembly of RNA-seq data in a Sheep Model for Delayed Bone Healing

Marten Jäger [1,2,5], Claus-Eric Ott [1,5], Johannes Grünhagen[1], Jochen Hecht[2], Hanna Schell[2,4], Stefan Mundlos[1,2,3], Georg N. Duda[2,4], Peter N. Robinson[1,2,3], Jasmin Lienau[2,4]

1) Institute for Medical Genetics, Charité-Universitätsmedizin Berlin, Augustenburgerplatz 1, 13353 Berlin, Germany
2) Berlin-Brandenburg Center for Regenerative Therapies (BCRT), Charité-Universitätsmedizin Berlin, Augustenburgerplatz 1, 13353 Berlin, Germany
3) Max Planck Institute for Molecular Genetics, Ihnestrasse 73, 14195 Berlin, Germany
4) Julius Wolff Institute and Center for Musculoskeletal Surgery, Charité-Universitätsmedizin Berlin, Augustenburgerplatz 1, 13353 Berlin, Germany
5) authors contributed equally to this work

Supplementary Figures and Tables

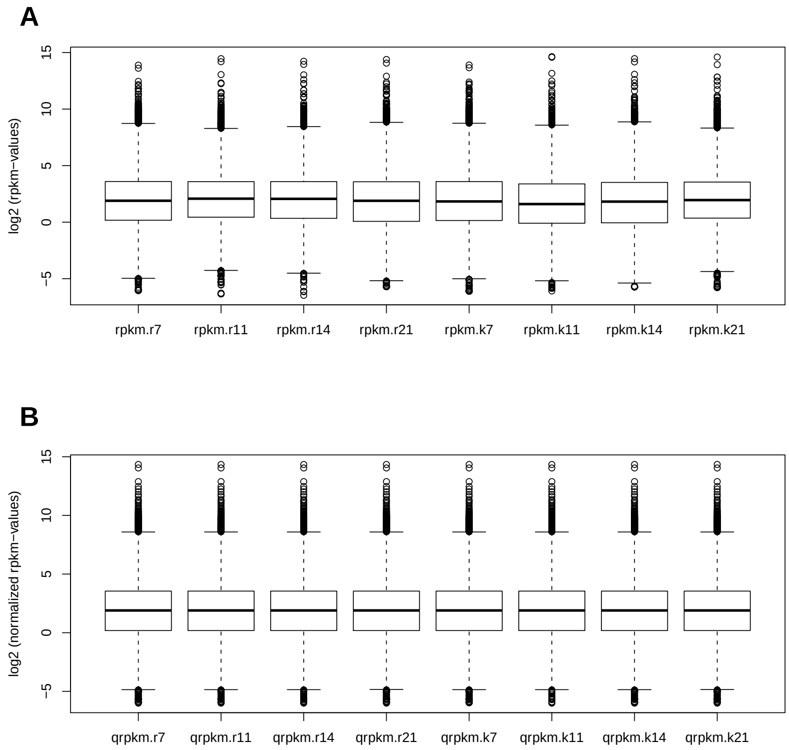

Figure S1. Boxplot of the RPKM values of the NGS data before (**A**) and after (**B**) quantile normalisation. r7–r21: Standard healing group, days 7–21. k7–k21: Delayed healing group, days 7–21.

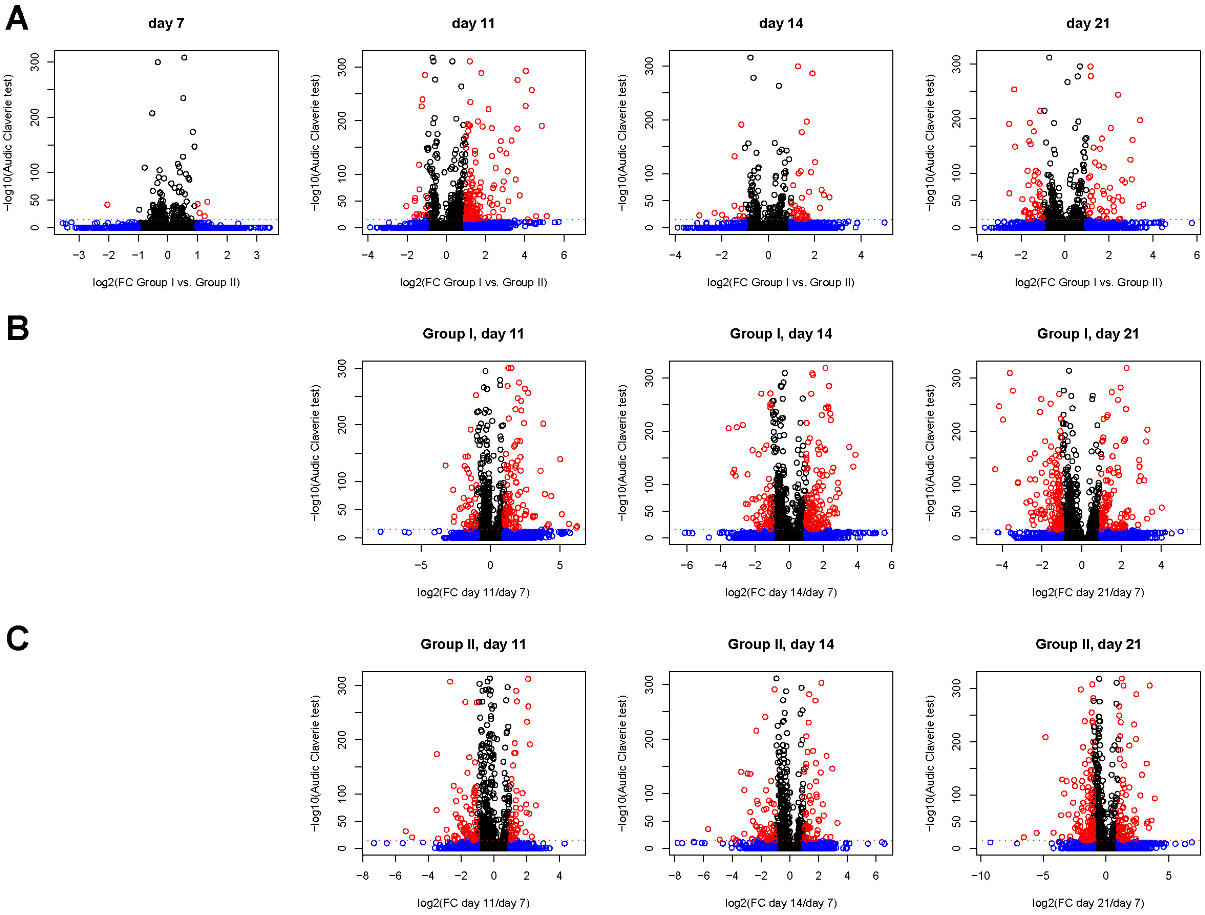

Figure S2. Volcano plots of the fold change based on normalized RPKM-values (x-axis) and the (BH) corrected p-values obtained from the Audic-Claverie Test (y-axis). **A** Direct comparison of Group I (standard healing) and Group II (delayed healing) at the indicated time points. **B** Time course of standard healing. **C** Time course of delayed healing. (B,C) The expression levels at the indicated time points were compared to the expression level at day 7. (A-C) The horizontal dotted red lines indicate the corrected p-value cut-off of $p < 10^{-15}$. An absolute fold change threshold > 2 was set for all analyses.

		Audic-Claverie (BH) corrected p-value			RPKM raw				RPKM quantile normalized			
GeneID	human.GeneSymbol	day 7 vs. 11	day 7 vs. 14	day 7 vs. 21	day 7	day 11	day 14	day 21	day 7	day 11	day 14	day 21
MYH7	MYH7	8.04e-09	1.08e-08	8.45e-09	4.45	48.172	127.092	33.327	4.411	51.208	132.848	33.353
MLRV	MYL2	1.17e-09	3.76e-10	1.92e-09	38.042	195.536	708.531	141.825	35.623	217.858	720.477	140.704
TNNT1	TNNT1	2.07e-119	0	6.51e-10	14.414	58.984	209.293	51.647	13.916	63.021	210.507	51.589
MYG	MB	9.44e-10	9.01e-10	1.89e-181	6.252	60.24	279.885	58.599	6.133	64.592	294.863	58.6
MYH2	MYH2	0	0	6.85e-10	2.73	24.566	63.156	21.138	2.729	25.583	64.107	20.84
TNNC2	TNNC2	1.65e-09	1.40e-09	1.15e-09	30.022	133.457	181.156	84.728	28.103	150.517	188.956	84.213
TNNT3	TNNT3	5.45e-257	0	2.43e-09	15.576	89.933	149.763	55.31	14.955	98.395	151.235	55.502
MYLPF	MYLPF	2.78e-08	0	8.46e-24	84.886	277.872	505.552	134.381	80.494	300.244	511.1	132.848
Q7M2Q7	COL9A1	1	1	3.93e-02	1.046	1.871	5.27	7.381	1.062	1.573	4.722	7.29
PPBT	ALPL	0	0	1.54e-09	6.753	56.92	74.998	49.155	6.606	61.03	76.831	48.729
SIAL	IBSP	0	0	6.55e-10	6.909	60.861	93.294	39.01	6.761	65.493	96.39	38.864
CO2A1	COL2A1	1.27e-08	0	0	11.503	17.912	153.692	231.634	11.138	18.098	156.127	236.162
Q2KJC7	POSTN	4.77e-301	0	0	60.577	126.857	221.017	150.779	57.212	140.965	222.124	150.203
COL1A1*	COL1A1	9.45e-06	0	1.52e-05	12519.457	18644.51	15791.9	17106.508	16939.966	16939.966	16939.966	16939.966
CD36	CD36	1	2.27e-146	0	54.813	52.499	100.696	166.172	51.368	55.779	104.57	164.703
CTSS	CTSS	3.38e-04	2.65e-30	5.33e-189	71.377	81.705	95.908	143.133	66.755	88.703	99.617	143.295
ITGAM	ITGAM	3.98e-01	2.56e-03	3.48e-08	26.797	30.614	22.241	37.945	25.415	32.471	22.791	37.713
Q2KIW2	CD68	1	4.16e-08	4.69e-08	67.281	73.609	94.58	185.91	63.069	78.927	97.788	184.945
MPEG1	MPEG1	1.35e-08	1	3.47e-131	49.479	62.931	48.103	84.769	45.766	67.819	50.056	84.301
Q29630	CD74	9.72e-35	1.15e-07	1.06e-07	258.746	320.229	206.66	453.375	241.344	338.336	209.77	451.973
GAPDH	GAPDH	2.13e-07	2.34e-07	7.90e-08	1054.04	473.163	386.404	366.507	971.522	531.41	405.678	369.573
HMOX1	HMOX1	6.81e-141	2.08e-07	0	401.19	279.946	140.169	100.126	364.945	305.928	144.235	99.682
PAI-1	PAI-1	6.59e-08	0	5.14e-08	380.037	72.2	42.464	33.159	338.336	77.4	44.245	33.173
IL1B	IL1B	4.90e-71	9.64e-10	2.01e-81	9.75	2.932	2.376	1.906	9.525	2.525	2.163	2.009
ADAMTS1	ADAMTS1	5.79e-223	3.69e-251	1.74e-08	34.46	16.529	15.078	10.686	32.05	16.598	15.041	10.501
A0JN60	TNC	6.29e-07	0	0	410.513	218.301	151.014	156.323	375.365	250.228	152.71	156.446

Table S1. RNA-SEQ results Group I (standard healing). (*) COL1A1 was among the highest expressed genes and had the same rank in all analyzed samples. Therefore the RPKM values are identical after quantile normalization.

		Audic-Claverie (BH) corrected p-value			RPKM raw				RPKM quantile normalized			
GeneID	human.GeneSymbol	day 7 vs. 11	day 7 vs. 14	day 7 vs. 21	day 7	day 11	day 14	day 21	day 7	day 11	day 14	day 21
MYH7	MYH7	1.46e-09	4.28e-09	3.65e-09	3.877	1.734	26.746	47.364	4.041	2.086	27.387	46.869
MLRV	MYL2	1.06e-34	4.60e-09	4.75e-09	16.317	1.763	137.739	349.241	15.441	2.122	141.572	344.38
TNNT1	TNNT1	1.30e-07	1.59e-09	1.38e-09	8.718	3.078	38.333	164.617	8.713	3.79	39.523	155.65
MYG	MB	5.36e-04	2.60e-146	0	6.416	2.544	49.946	269.558	6.582	3.127	51.693	262.055
MYH2	MYH2	2.13e-22	3.17e-09	3.33e-09	3.151	1.008	22.062	93.643	3.298	1.218	22.524	91.435
TNNC2	TNNC2	4.58e-57	9.05e-09	8.50e-09	36.853	6.164	86.516	452.204	33.219	7.301	89.177	436.98
TNNT3	TNNT3	1.90e-24	5.18e-09	5.14e-09	14.654	3.765	68.597	287.326	13.97	4.632	72.318	279.541
MYLPF	MYLPF	2.98e-174	1.01e-08	1.14e-08	75.891	5.194	206.623	802.254	68.7	6.256	208.591	736.568
Q7M2Q7	COL9A1	1	1.21e-11	9.71e-12	0.399	0.239	36.081	50.222	0.428	0.325	36.714	49.653
PPBT	ALPL	0	5.75e-10	8.06e-10	7.165	62.105	52.368	28.845	7.32	66.125	54.046	28.779
SIAL	IBSP	1.61e-09	2.35e-09	2.20e-09	3.41	27.292	61.399	25.195	3.571	29.281	64.31	25.16
CO2A1	COL2A1	4.90e-08	1.13e-08	1.23e-08	10.204	14.001	895.031	886.028	10.113	15.411	979.278	806.205
Q2KJC7	POSTN	1	0	0	49.959	57.271	224.748	328.361	45.53	60.848	228.255	314.657
COL1A1*	COL1A1	0	3.14e-05	1	12904.998	24526.308	18437.221	15588.83	16939.966	16939.966	16939.966	16939.966
CD36	CD36	2.10e-33	2.16e-15	1.13e-02	29.59	16.767	19.993	24.201	27.195	18.064	20.538	24.164
CTSS	CTSS	1.44e-60	7.22e-06	4.50e-08	46.622	23.681	37.218	32.856	42.303	25.491	38.146	32.91
ITGAM	ITGAM	1.86e-03	4.49e-09	1.64e-27	20.035	15.916	11.524	11.364	18.747	17.185	11.816	11.324
Q2KIW2	CD68	3.70e-15	1	1	52.05	34.859	44.587	54.534	47.442	37.196	45.968	53.527
MPEG1	MPEG1	9.64e-20	3.55e-08	2.07e-23	41.472	31.194	26.09	30.286	37.624	33.518	26.596	30.233
Q29630	CD74	0	0	7.01e-08	308.232	136.623	120.265	170.16	279.541	150.203	124.781	161.887
GAPDH	GAPDH	8.63e-151	0	0	1117.96	791.766	612.4	553.399	979.278	921.312	653.303	514.791
HMOX1	HMOX1	1.21e-07	2.55e-07	0	255.39	156.385	184.827	78.103	233.383	175.522	185.53	76.539
PAI-1	PAI-1	0	1.57e-08	0	312.527	172.292	147.551	65.719	287.882	189.531	149.962	64.782
IL1B	IL1B	1.54e-09	6.29e-10	2.18e-09	5.783	2.661	2.566	2.39	5.939	3.263	2.796	2.18
ADAMTS1	ADAMTS1	8.46e-01	8.08e-08	2.06e-184	38.96	36.031	32.636	19.624	35.061	38.239	33.4	19.664
A0JN60	TNC	1.23e-06	1	1	276.134	462.574	307.102	267.49	255.743	543.253	314.657	259.311

Table S2. RNA-SEQ results Group II (delayed healing). (*) COL1A1 was among the highest expressed genes and had the same rank in all analyzed samples. Therefore the RPKM values are identical after quantile normalization.

		Group I (standard healing)			Group II (delayed healing)			FC delayed versus standard healing			
GeneID	human.GeneSymbol	day 7 vs. 11	day 7 vs. 14	day 7 vs. 21	day 7 vs. 11	day 7 vs. 14	day 7 vs. 21	day 7	day 11	day 14	day 21
MYH7	MYH7	11.61	30.119	7.562	0.516	6.777	11.598	0.916	0.041	0.206	1.405
MLRV	MYL2	6.116	20.225	3.95	0.137	9.168	22.303	0.433	0.01	0.196	2.448
TNNT1	TNNT1	4.529	15.127	3.707	0.435	4.536	17.864	0.626	0.06	0.188	3.017
MYG	MB	10.532	48.08	9.555	0.475	7.854	39.813	1.073	0.048	0.175	4.472
MYH2	MYH2	9.376	23.494	7.638	0.369	6.831	27.728	1.208	0.048	0.351	4.387
TNNC2	TNNC2	5.356	6.724	2.997	0.22	2.685	13.155	1.182	0.049	0.472	5.189
TNNT3	TNNT3	6.579	10.113	3.711	0.332	5.177	20.01	0.934	0.047	0.478	5.037
MYLPF	MYLPF	3.73	6.35	1.65	0.091	3.036	10.722	0.853	0.021	0.408	5.544
Q7M2Q7	COL9A1	1.481	4.447	6.864	0.76	85.865	116.127	0.403	0.207	7.775	6.811
PPBT	ALPL	9.238	11.63	7.376	9.034	7.384	3.932	1.108	1.083	0.703	0.591
SIAL	IBSP	9.686	14.256	5.748	8.201	18.011	7.046	0.528	0.447	0.667	0.647
CO2A1	COL2A1	1.625	14.017	21.203	1.524	96.834	79.72	0.908	0.851	6.272	3.414
Q2KJC7	POSTN	2.464	3.882	2.625	1.336	5.013	6.911	0.796	0.432	1.028	2.095
CD36	CD36	1.086	2.036	3.206	0.664	0.755	0.889	0.529	0.324	0.196	0.147
CTSS	CTSS	1.329	1.492	2.147	0.603	0.902	0.778	0.634	0.287	0.383	0.23
ITGAM	ITGAM	1.278	0.897	1.484	0.917	0.63	0.604	0.738	0.529	0.518	0.3
Q2KIW2	CD68	1.251	1.55	2.932	0.784	0.969	1.128	0.752	0.471	0.47	0.289
MPEG1	MPEG1	1.482	1.094	1.842	0.891	0.707	0.804	0.822	0.494	0.531	0.359
Q29630	CD74	1.402	0.869	1.873	0.537	0.446	0.579	1.158	0.444	0.595	0.358
GAPDH	GAPDH	0.547	0.418	0.38	0.941	0.667	0.526	1.008	1.734	1.61	1.393
HMOX1	HMOX1	0.838	0.395	0.273	0.752	0.795	0.328	0.64	0.574	1.286	0.768
PAI-1	PAI-1	0.229	0.131	0.098	0.658	0.521	0.225	0.851	2.449	3.389	1.953
IL1B	IL1B	0.265	0.227	0.211	0.549	0.471	0.367	0.623	1.292	1.293	1.085
ADAMTS1	ADAMTS1	0.518	0.469	0.328	1.091	0.953	0.561	1.094	2.304	2.221	1.873
A0JN60	TNC	0.667	0.407	0.417	2.124	1.23	1.014	0.681	2.171	2.06	1.658

Genes selected as endogenous controls for qPCR analyses

GTPB1	GTPBP1	0.995	0.916	0.971	1.167	0.988	0.991	0.972	1.141	1.048	0.992
A5PJZ7	HDAC6	1.028	0.913	1.008	1.114	0.937	0.928	1.014	1.099	1.041	0.934
RSMN	SNRPN	1.062	1.071	0.915	1.103	1.101	1.092	0.942	0.978	0.968	1.125

Table S3. RNA-SEQ derived foldchanges. Expression levels relative to day 7 in Group I (standard healing), relative to day 7 in Group II (delayed healing), and ratios of the expression levels at the indicated time points are shown.

Gene	Group I (standard healing)				Group II (delayed healing)				Fold change delayed versus standard healing			
	day 7	day 11	day 14	day 21	day 7	day 11	day 14	day 21	day 7	day 11	day 14	day 21
MYH7	1.000	15.135	46.212	11.141	1.000	0.229	34.568	78.072	0.395	0.006	0.295	2.765
MLRV	1.000	4.533	16.054	3.581	1.000	0.112	14.382	44.842	0.247	0.006	0.221	3.090
TNNT1	1.000	3.826	17.265	3.931	1.000	0.134	9.670	47.151	0.346	0.012	0.194	4.147
MYG	1.000	13.568	96.311	17.614	1.000	0.111	15.904	94.366	1.123	0.009	0.185	6.014
MYH2	1.000	8.092	14.483	4.678	1.000	0.036	9.657	30.466	1.311	0.006	0.874	8.535
TNNC2	1.000	5.859	9.387	2.927	1.000	0.022	6.965	31.808	0.710	0.003	0.527	7.714
TNNT3	1.000	4.450	11.234	3.238	1.000	0.190	6.766	21.994	0.854	0.036	0.514	5.801
MYLPF	1.000	3.349	4.828	1.429	1.000	0.039	4.240	16.559	0.581	0.007	0.510	6.729
COL9A1	1.000	1.486	228.149	395.156	1.000	0.249	640.279	1005.252	2.899	0.485	8.135	7.374
PPBT	1.000	14.237	21.846	13.740	1.000	5.536	13.785	6.593	1.060	0.412	0.669	0.509
SIAL	1.000	12.919	13.020	11.964	1.000	6.778	15.980	10.611	0.774	0.406	0.950	0.686
COL2A1	1.000	3.969	1274.519	2148.074	1.000	3.859	5453.677	8859.409	1.569	1.526	6.715	6.472
Q2KJC7	1.000	1.979	2.096	2.467	1.000	0.971	2.329	4.368	1.252	0.615	1.391	2.217
COL1A1	1.000	1.682	2.047	2.084	1.000	1.604	1.882	2.256	1.058	1.009	0.973	1.145
CD36	1.000	0.864	1.133	2.890	1.000	0.880	0.286	0.441	0.814	0.830	0.205	0.124
CTSS	1.000	1.179	1.006	2.040	1.000	0.991	0.697	0.957	0.711	0.598	0.493	0.334
ITGAM	1.000	1.359	0.974	2.060	1.000	0.708	0.579	0.553	0.889	0.463	0.529	0.239
CD68	1.000	1.169	1.250	2.673	1.000	0.784	0.631	0.621	1.037	0.695	0.523	0.241
MPEG1	1.000	0.961	1.078	1.789	1.000	0.859	0.629	0.744	0.993	0.888	0.580	0.413
CD74	1.000	1.325	0.913	1.869	1.000	0.489	0.390	0.441	1.188	0.439	0.507	0.280
GAPDH	1.000	0.521	0.436	0.373	1.000	0.777	0.790	0.486	0.939	1.399	1.699	1.221
HMOX1	1.000	0.461	0.213	0.272	1.000	0.502	0.444	0.224	0.673	0.733	1.399	0.555
PAI-1	1.000	0.210	0.139	0.115	1.000	0.512	0.342	0.132	1.113	2.708	2.740	1.284
IL1B	1.000	0.161	0.060	0.075	1.000	0.718	0.311	0.114	0.644	2.866	3.348	0.989
ADAMTS1	1.000	0.416	0.399	0.296	1.000	1.189	1.201	1.014	0.701	2.005	2.109	2.397
A0JN60	1.000	0.452	0.477	0.463	1.000	1.482	1.122	1.463	0.701	2.301	1.651	2.215

Endogenous Controls												
GTPB1	1.000	1.144	1.061	1.157	1.000	0.893	0.787	0.687	1.377	1.075	1.022	0.817
HDAC6	1.000	1.095	0.913	0.933	1.000	0.919	0.851	0.826	0.994	0.835	0.926	0.881
SNRPN	1.000	0.798	1.032	0.926	1.000	1.218	1.494	1.762	0.730	1.115	1.057	1.390

Table S4. qPCR validation of selected genes. Expression levels relative to day 7 in Group I (standard healing), relative to day 7 in Group II (delayed healing), and ratios of the expression levels at the indicated time points are shown. Stably expressed genes in the RNA-seq analyses were used as endogenous controls and averaged. Down-regulation larger than 2-fold (value<0.5) is indicated in dark grey, up-regulation larger than 2-fold (value>2.0) in light grey.

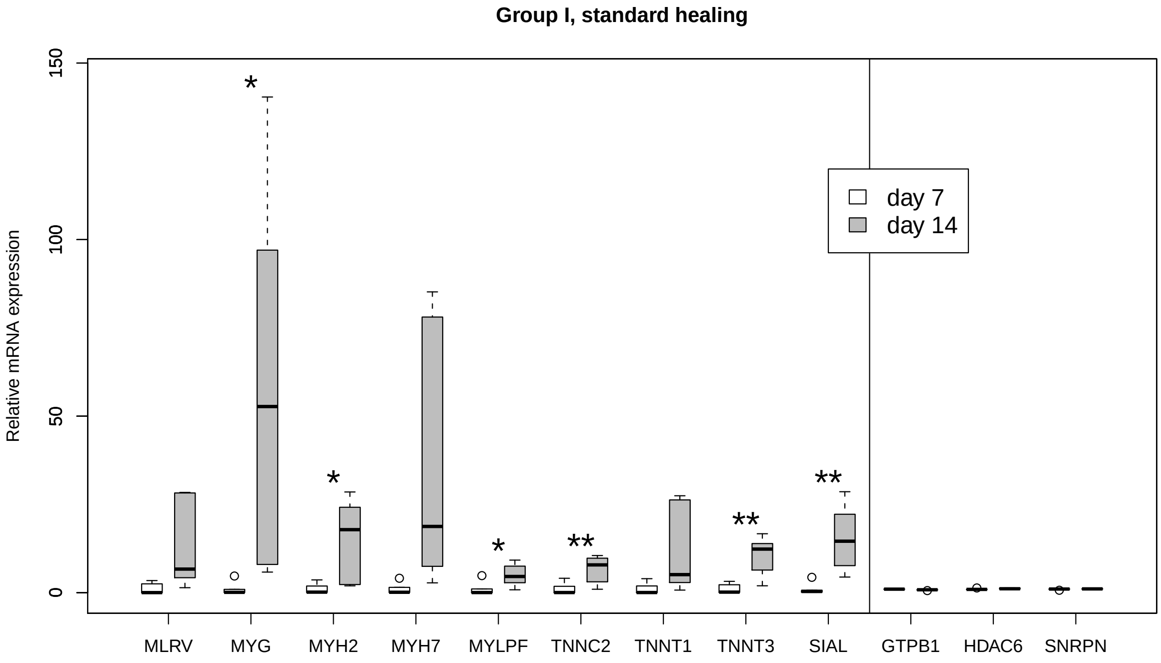

Figure S3. Q-PCR investigation of selected genes in individual samples of animals from group I (standard healing; n=6 for day 7 and n=6 for day 14). All samples were run in triplicates in separate tubes to permit the quantification of the target genes' mRNA expression relative to the mean expression of *GTPB1*, *HDAC6* and *SNRPN*, i.e. three stably and highly expressed genes identified in the RNA-seq data. * $p < 0.05$,** $p < 0.02$.
